# Allergic contact dermatitis to isothiazolinones in a rubber factory: A case report

**DOI:** 10.1002/ccr3.6186

**Published:** 2022-08-03

**Authors:** Anouare Hrairi, Nada Kotti, Massara Baklouti, Emna Bahloul, Imen Sellami, Feriel Dhouib, Kaouthar Jmal Hammami, Mohamed Larbi Masmoudi, Hamida Turki, Mounira Hajjaji

**Affiliations:** ^1^ Occupational Department and Health Disease, Hedi Chaker University Hospital University of Sfax Sfax Tunisia; ^2^ Dermatology Department, Hedi Chaker University Hospital University of Sfax Sfax Tunisia

**Keywords:** allergic contact dermatitis, methylchloroisothiazolinone, methylisothiazolinone, occupational, rubber

## Abstract

Isothiazolinones, used as preservative, are known to be skin sensitizers. Although cosmetics represent their main source, occupational exposure may be a significant origin of eczema. While allergic eczema related to these derivates have been reported in a number of professional sectors, their presence in the same workplace was not common.

## Abstract 2

Isothiazolinones are commonly known as skin sensitizers in both domestic and workplace areas. Rubber manufacturing should be considered as a possible occupational exposure to isothiazolinones. This consideration will facilitate further declaration and recognition of allergic dermatitis related to these substances in this specific workplace.

## 1 INTRODUCTION

Isothiazolinones are heterocyclic compounds characterized by a nitrogen and sulfur aromatic ring (1, 2 ‐thiazol ‐ 3 ‐one). This activated N‐S bond provides an antimicrobial activity to these molecules, but it also has an inherent capability to cause sensitization.^1^, ^2^ Isothiazolinones' derivates frequently used are methylchloroisothiazolinone (MCI), methylisothiazolinone (MI), benzisothiazolinone (BIT), and octylisothiazolinone (OIT). These preservatives differ in potency of sensitization: MCI > MI > BIT> OIT consistent with their extent of use.^3^, ^4^ The best known is a mixture of (MCI) and (MI) often named by its most common trade name Kathon™. This preservative has been shown to be effective at very low concentrations with microbiocide activity against a wide spectrum of fungi as well as, Gram‐positive and Gram‐negative bacteria.^5^ It has been used since the early 1980s as a preservative in cosmetics.^6^ This exposure was responsible for first cases of contact dermatitis from Kathon® CG in 1984.^7^ Although cosmetics represent the main source of sensitization to MCI/MI and MI,^8^ these allergens are frequently responsible for occupational eczema. Between 2008 and 2013, cases of occupational contact allergy to MCI / MI or MI increased six‐fold^4^ and reached 16.8% in 2015.^9^ Various sources of exposure to isothiazolinone derivatives in the workplace were identified such as water‐based paints, cutting oils, glues, latex emulsions, and papermills.^10^

Occupational exposure to these allergens is usually described in smaller case reports or series.^11^, ^12^, ^13^, ^14^ Thus, we present a case of a worker in a rubber factory presenting an allergic contact dermatitis to MCI/MI and MI exacerbated by his professional activity.

## 2 CLINICAL FEATURES

**FIGURE 1:**
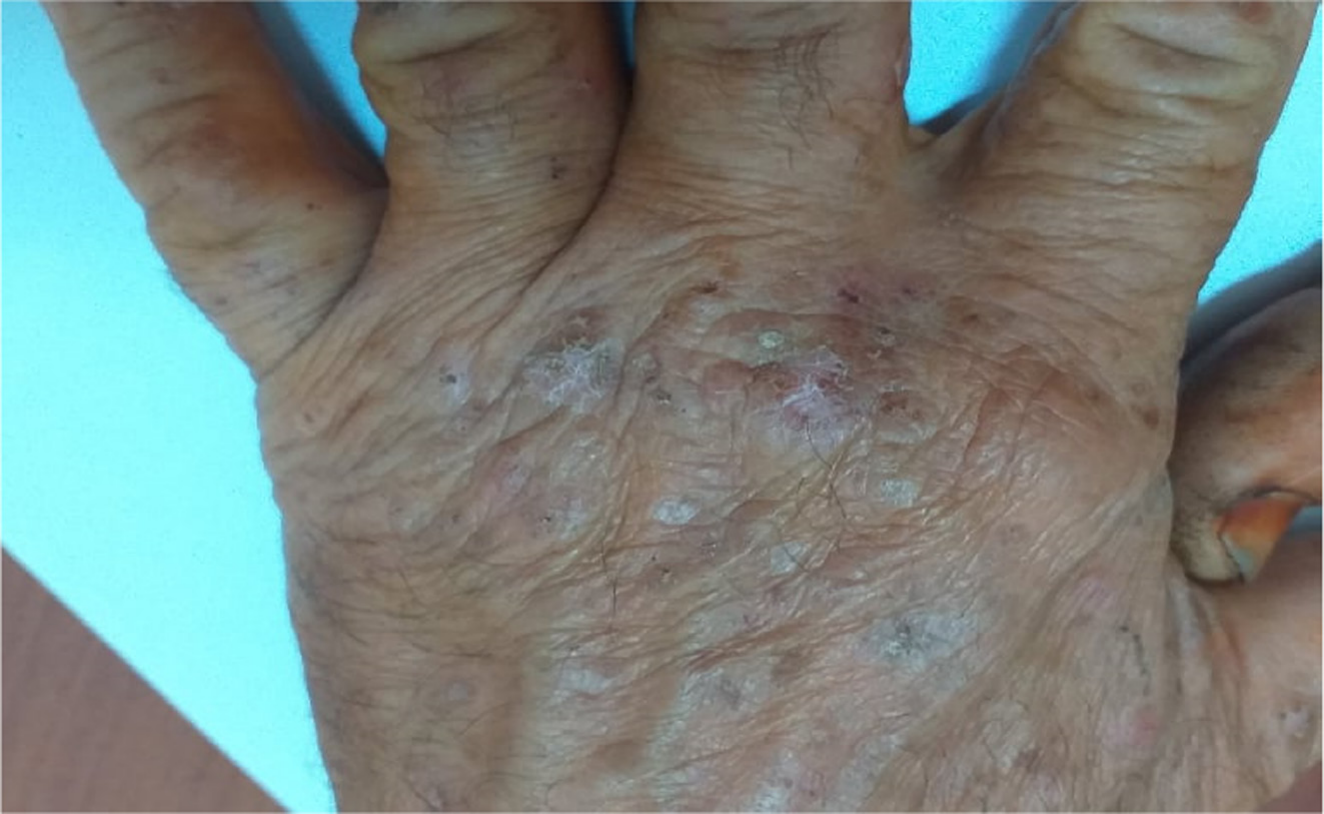
Erythematosquamous lesions on hands

**FIGURE 2:**
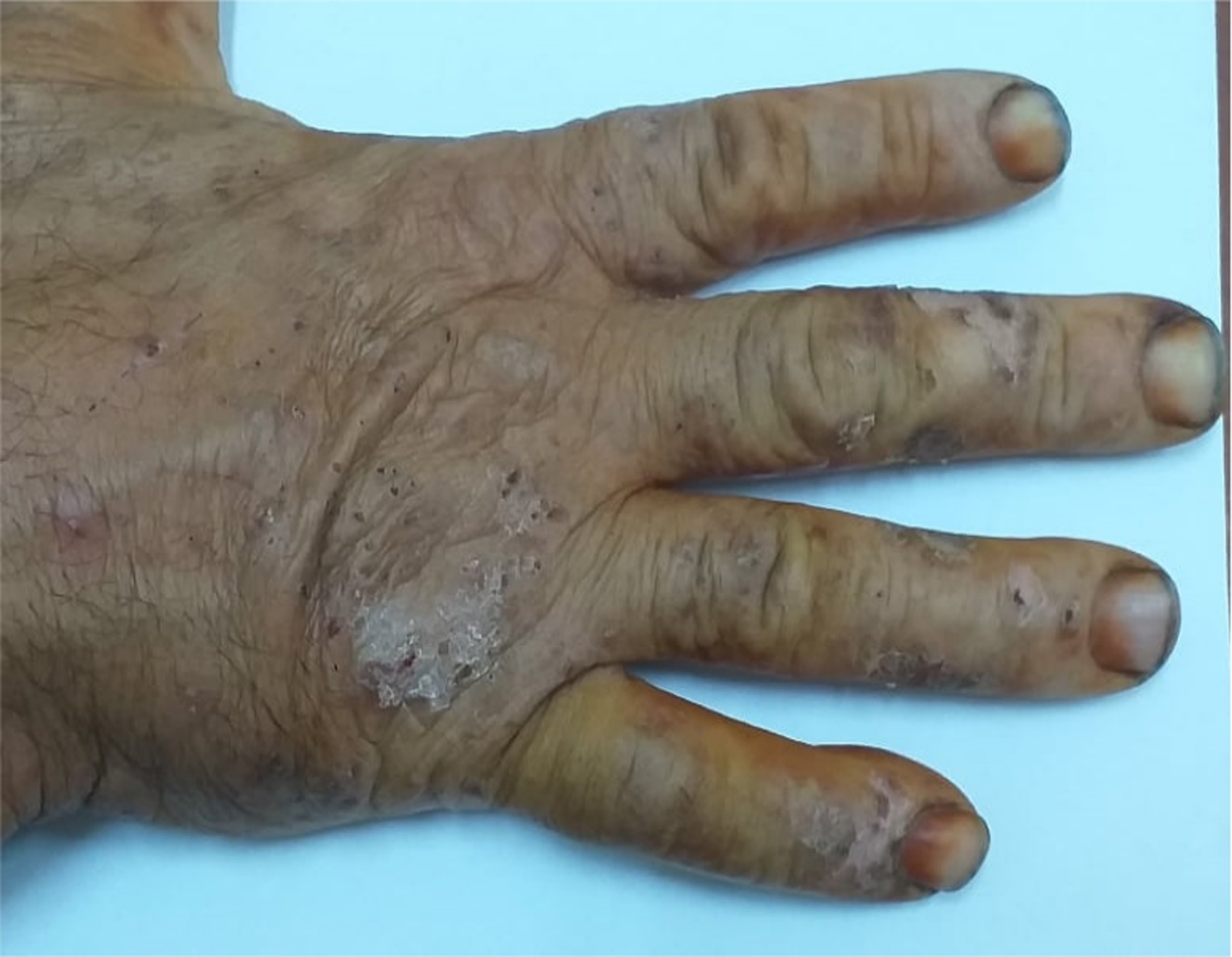
Erythematosquamous lesions on hands

**FIGURE 3:**
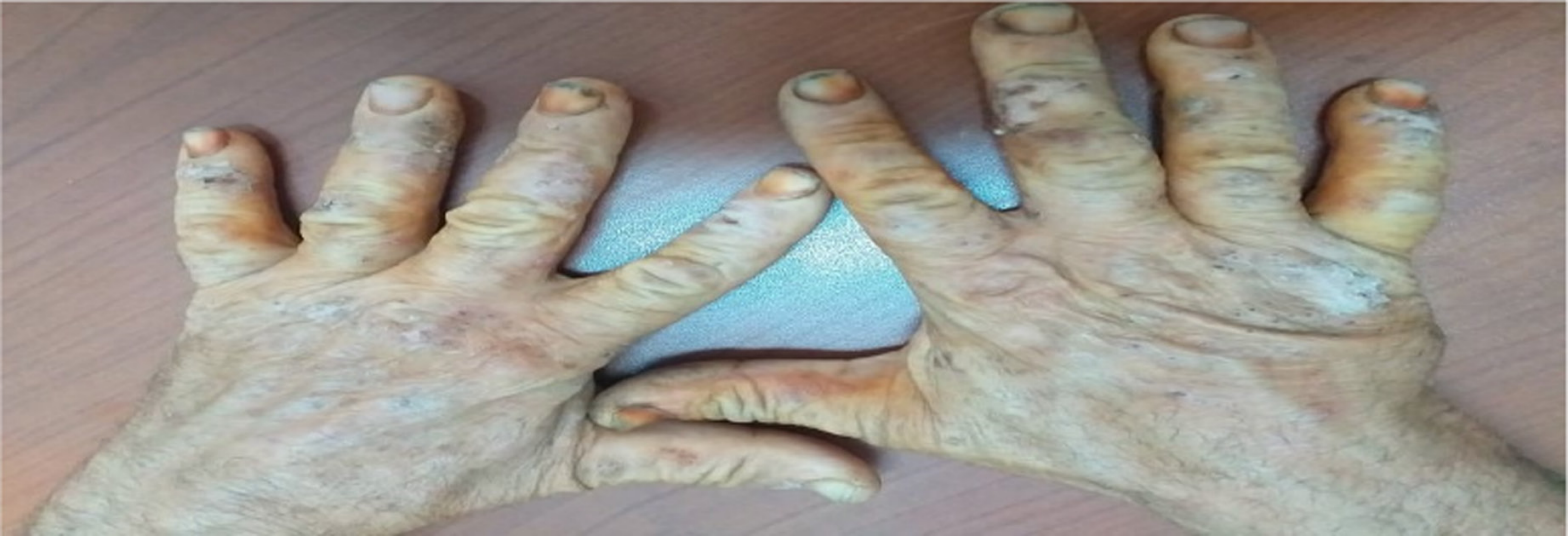
Erythematosquamous lesions on hands

A 55‐year‐old male patient with no history of previous skin disease was referred to our occupational department in 2020 presenting an allergic contact dermatitis evolving since 2 months probably related to his professional activity after the introduction of new products. Clinically at the acute phase, he presented vesicular and exudative itchy lesions on both hands. Then, this eczema had run a chronic relapsing course after discontinuation of dermocorticoids with appearance of intensely pruriticerythematosquamous lesions (Figures 1, 2, 3). Chronologic evolution shows association with the workplace environment: Aggravation of symptoms while working and their attenuation during periods off‐work. With this dermatitis, the worker had no associated respiratory or general symptoms.

Epicutaneous test was performed with chemotechnique European baseline series. It was applied on the upper back using Finn Chambers®. Positive reactions to MCI/MI (2+) MI (3+), methyl dibromoglutaronitrile (2+), and Textile dye mix (3+) were found at the 48 h reading. We had not test the patient with other rubber additives than those included in the baseline series neither with other series regarded to the persistence and chronic evolution of dermatologic lesions objectified during different consultations.

## 3 JOB CHARACTERISTICS

These were assessed according to the patient description and an inventory of the source materials and substances used in the factory. The patient had brought to our department the products used and the correspondent technical and safety data sheets. Moreover, the type of adhesive used and the heat resistant gloves were brought too and this helped as to recognize their exact composition. Further information was not available from the manufacturer.

Since 2006, the patient has been working in manufacturing of automotive rubber products. The substances used were elastomeric crumb rubber (particles or powder): (Natural rubber (NR), Styrene‐butadiene rubber (SBR), Ethylene‐propylene rubber (EPM and EPDM) or Nitrile rubber (NBR)); Additives for vulcanization (sulfur, Thiuram or Thiazole derivatives) and glues. According to the patient, the (SBR) was recently introduced in the factory before the onset of the lesions.

An emulsion polymerization process for latex was used. The patient interfered while the molding machine was progressing with manual loading or unloading, wearing heat resistant gloves made of tanned leather. He was charged in filling out the container with elastomeric particles. This operation generated a lot of dust while using powdered elastomeric crumb rubber. The exposure was not insignificant regarding the absence of appropriate local exhaust ventilation. The finishing of the product was processed by brushing it with a wire brush. Then, he applied the primer (Chemosil® 211) and the adhesive (Chemosil® 225) to the article without wearing proper protection.

Prior to current skin manifestations, the patient reported unexplored similar reactions. A re‐evaluation of the case history revealed that 14 years previously, the patient probably developed an episode of hands eczema while he was working as a painter (he used to manipulate water‐based paint without wearing proper protection).

## 4 DISCUSSION

The patient presented skin sensitization (defined as an immunological response to previous exposure to a substance which results in an inflammatory skin reaction) and had positive reactions to MCI/MI (2+) and MI (3+) when patch tested. Test positivity to MCI/MI and MI may represent two separate sensitizations due to a co‐exposure since both of them were classified in the 1A category as skin sensitizers according to the Commission Regulation (EU) in 2018.^15^ In addition, given chemical similarities between the isothiazolinone derivatives, cross‐reactivity may be discussed too. In fact, several studies suggest that skin‐sensitization to one of these allergens is likely to cause positive reaction to the other in the patch test.^16^ According to Geirer et al, given immunological cross‐reactions, the increase in primary sensitization to MI from 2009 to 2011 explained the rise in MCI/MI reactions.^17^

In our case, extra professional explanations to isothiazolinones' sensitization were excluded such as the use of a new product containing a biocide especially in cosmetic products and detergents or a pesticide in his gardening activity. Therefore, this sensitization is probably related to job activity, in which the patient was exposed to rubber, leather, and adhesive.

In terms of occupational exposure to MCI/MI, painters seem to constitute the most significant subgroup.^18^ Based on the case history, the first episode of eczema occurred in a paint manufactory where the patient was directly exposed to water‐based paint.

The primary sensitization to isothiazolinones is most likely related to the exposure to water‐based paint. The patient had developed further the allergic contact dermatitis while working in the rubber factory.

Since 2006, the patient worked as a machine operator in the rubber factory. Despite widespread automation in the rubber industry, certain workers are at an increased risk of becoming sensitized to rubber additives. Among rubber workers, those who weigh the various ingredients which are added to precut chunks of natural or synthetic rubber in large mixers (Banbury mixers) and skilled laborers involved in the fabrication of tires are most likely to develop allergic sensitization. Those operating the Banbury mixers, the calenders, the extruders, and the molding machines, are also at some risk, as are those workers involved in packing and shipping the final product.^16^ Rubber industry (latex emulsions) represents one of the most important non‐cosmetic sources of isothiazolinones derivates.^10^ The exposure to Kathon™ in the rubber factory may be due to the manipulation of elastomeric crumb rubber (especially the SBR containing this preservative) without proper protection. In fact, according to the safety and technical data sheets of this product, it is recommended for the control of bacteria and fungi in the manufacture and storage of synthetic and natural polymer latices including styrene/butadiene intended for industrial use.^19^, ^20^ This explains the fact that the introduction of SBR had trigged skin lesions in patient's hands.

As mentioned above, the patient wore heat resistant gloves made of tanned leather during the filling operation. This could result in additional exposure to isothiazolinones derivates (MCI/MI and BIT), which are used as preservative in leather industry. Sensitization is possible for those working in the manufacturing and processing of leather or even after exposure to leather goods containing these biocides.^16^, ^21^ Allergic dermatitis caused by leather gloves is most commonly related to the chromium salts used in the tanning of leather.^22^ Yet, the patch test was negative to potassium dichromate in the standard series.

Manipulation of adhesives and glues (Chemosil® 211 and Chemosil® 225) in the workplace represents a supplementary source of exposure to isothiazolinones.^23^, ^24^ But, according to safety data sheet, those products did not contain isothiazolinones derivates and contain tetrachloroethylene.^25^, ^26^, ^27^ According to a study in UK, adhesives requiring the solvent tetrachloroethylene had lower isothiazolinone content because they are less water soluble and therefore likely to have less need for preservatives such as isothiazolinones.^28^

An important isothiazolinone derivate BIT has not been tested with the European baseline series, therefore sensitization to this allergen cannot be eliminated. Separate sensitization to both MIT and BIT is probable rather than a cross‐reaction. Geier et al have demonstrated that less than 10% of sensitized patients to MI have positive reaction to BIT.^29^, ^30^ In fact, this derivate is considered as an important sensitizer in both industrial and household products, such as cleaning and impregnating agents, paints, polishes, and printing inks. In 2012, it was rejected by the European scientific Committee on Consumer Safety for use in cosmetic product, owing to the risk of sensitization.^4^, ^31^ A primary exposure to BIT remains possible while the patient was working in a paint manufactory. According to a multicenter study of paints from five European countries, MIT and BIT are both widely used in paint.^32^ Moreover, BIT represents a biocide frequently used in rubber factory,^33^ wherein the recycled elastomeric particles contain SBR, the biocidal agent used is N‐butyl‐1,2‐benzisothiazo lin‐3‐one.^34^

Regarded the probable incrimination of BIT in the generation of hand eczema, we have considered this dermatitis as an occupational disease according to Tunisian legislation (BIT exists in Table 59 of occupational diseases) even if this allergen was not tested with the European guideline series.

Finally, it has to be noted that, exposure to heat and wearing gloves made by rough leather may worsen skin manifestation by causing friction, profuse sweating of the hands and maceration. Thus, in this context, wearing this type of gloves is not recommended because sensitization to leather preservative as isothiazolinones derivates remains possible, and even so, it can contribute to the worsening of hand eczema. Besides, organic solvents present in adhesives manipulated by the patient may cause irritant occupational dermatitis.^35^ Therefore, numerous measures were recommended to the employer such as the provision of protective equipment (replace leather gloves with other type suitable for heat exposure, chemical‐resistant protective gloves [EN 374] while the manipulation of adhesive and facial protection: safety goggles or face shield) and adequate ventilation in the workshop.

## 5 CONCLUSION

Rubber factory may be a source of occupational dermatitis due to different exposures in the workplace. Having hand eczema may have severe consequences such as a career change or even job loss. Occupational doctor should be aware of these consequences in order to preserve patients' jobs and to improve their working life quality. This awareness requires more knowledge about the broad range of occupational products which may trigger a hand contact dermatitis and setting strict protective measures in the workplace. Therefore, further researches are needed in this field to identify different causal allergens.

## AUTHOR CONTRIBUTIONS

A. Hrairi, N. Kotti, M.Baklouti, and E. Bahloul involved in concept, design, and drafting of the study. I.Sellami and F. Dhouib involved in bibliography. K. Jmal Hammami, M L. Masmoudi, and M. Hajjaji revised and approved of final manuscript.

## CONFLICT OF INTEREST

All authors have nothing to declare. They know of no conflicts of interest associated with this publication, and there has been no significant financial support for this work that could have influenced its outcome.

## CONSENT

Written informed consent was obtained from the patient to publish this report in accordance with the journal's patient consent policy.

## Data Availability

The data availability statement is available
